# Sex- and Gender-Based Analysis in Cannabis Treatment Outcomes: A Systematic Review

**DOI:** 10.3390/ijerph17030872

**Published:** 2020-01-30

**Authors:** Andreea C. Brabete, Lorraine Greaves, Natalie Hemsing, Julie Stinson

**Affiliations:** 1 Centre of Excellence for Women´s Health, E311-4500 Oak Street, Vancouver, BC V6H 3N1, Canada; lgreaves@cw.bc.ca (L.G.); nhemsing@cw.bc.ca (N.H.); juliestinson7@gmail.com (J.S.); 2 School of Population and Public Health, University of British Columbia, Vancouver, BC V6T 1Z4, Canada

**Keywords:** sex- and gender-based analysis, SGBA, cannabis use disorder, randomized controlled trial

## Abstract

There is evidence that sex- and gender-related factors are involved in cannabis patterns of use, health effects and biological mechanisms. Women and men report different cannabis use disorder (CUD) symptoms, with women reporting worse withdrawal symptoms than men. The objective of this systematic review was to examine the effectiveness of cannabis pharmacological interventions for women and men and the uptake of sex- and gender-based analysis in the included studies. Two reviewers performed the full-paper screening, and data was extracted by one researcher. The search yielded 6098 unique records—of which, 68 were full-paper screened. Four articles met the eligibility criteria for inclusion. From the randomized clinical studies of pharmacological interventions, few studies report sex-disaggregated outcomes for women and men. Despite emergent evidence showing the influence of sex and gender factors in cannabis research, sex-disaggregated outcomes in pharmacological interventions is lacking. Sex- and gender-based analysis is incipient in the included articles. Future research should explore more comprehensive inclusion of sex- and gender-related aspects in pharmacological treatments for CUD.

## 1. Introduction

Growing evidence related to the importance of sex- and gender-based factors within health research has led to increased interest among researchers, funding agencies, scientific journals and database creators to find innovative ways of examining these factors in previously unexplored areas [1,2,3]. The integration of sex- and gender-related factors into research, policy, or health programs revisits or identifies the influence of components such as anatomy, physiology, genetics and other bodily characteristics biological (sex-based) and the social and cultural milieu affecting humans socio-cultural (gender-based) is known as sex- and gender-based analysis (SGBA) [4]. Sex and gender are not independent of other social characteristics and they might interact with each other and other characteristics to influence health outcomes [5].

Randomized controlled trials (RCT) provide the strongest research evidence and are often used to test the efficacy of new pharmacological interventions. However, sex- and gender-based analysis in RCTs is very scarce. For example, in a study that analyzed 100 Canadian-led or funded RCTs, Welch et al. found that 98% of studies included sex in the description of sociodemographic characteristics of the participants, while only 6% conducted a subgroup analysis across sex, and only 4% reported sex-disaggregated data. None of the examined articles included a definition of “sex” or “gender” nor a comprehensive sex- and gender-based analysis [6]. Failing to include a sex- and gender-based analysis of the outcomes might have important and serious clinical consequences for individuals or subgroups of patients.

There are differences between women and men in referrals and pathways to substance use treatment in general. For example, women are less likely to be referred to residential treatment than men [7]; women are more likely to be referred to outpatient treatment vs. residential treatment [7,8]. Women tend to access substance use services via primary health care or mental health services vs. specialty substance use treatment services [8,9], while men are more likely to enter treatment via the criminal justice system [10]. Lack of awareness of options, stigma, confrontational treatment models, and lack of childcare are some of the common barriers encountered by women when accessing treatments for substance use [9]. Women tend to enter treatment with a more severe clinical profile and more problems related to mental health, family, interpersonal relationships, and physical health [9,10,11,12]; while men have more legal, criminal, and financial problems [13].

There are also differences in response to treatment for other substance use. For example, evidence derived using a sex- and gender-based analysis reveals that women have additional difficulties in tobacco smoking cessation. Women have poorer smoking cessation outcomes with some pharmacological supports, including nicotine replacement therapy, regardless of whether combined with counselling [14]; and buproprion [15]. In contrast, treatment with varenicline has shown similar, or better, outcomes for women compared to men [16,17,18]. Women tend to require more smoking quit attempts before achieving cessation. While women report lower quit rates, the use of any medication increases women’s likelihood of cessation [19].

Women and men receiving treatment for alcohol use disorder (AUD) report similar rates in reductions and/or abstinence from alcohol, including medical management and behavioral counselling for AUD [20]; treatment with the medication acamprosate (based on a meta-analysis) [21]; and residential treatment [22]. Studies on the effectiveness of naltrexone treatment for AUD treatment are mixed, with some studies reporting similar outcomes for women and men [22,23], and others reporting a greater reduction in craving scores for women [24], or greater reductions in alcohol use (and other substance use) in men [25]. The limited evidence examining sex differences in treatment outcomes for opioid use disorder (OUD) have reported similar improvements in opioid use outcomes for women and men following a medical management intervention (tapering with buprenorphine–naloxone) either alone or combined with counselling [26].

## 2. Sex- and Gender-Based Analysis in Cannabis Research

There is growing evidence that sex- and gender-related factors are involved in cannabis patterns of use, health effects and biological mechanisms. Men and boys are more likely to initiate cannabis use earlier, and use more frequently and in greater quantities, compared to women and girls. However, the gender gap has been narrowing over time [27,28]. For example, an analysis of US trends in adolescent cannabis use from 1999 to 2009 revealed that in 1999, 51% of boys and 43.4% of girls reported lifetime cannabis use, while in 2013, this decreased to 42.1% for boys and 39.2% for girls [27]. Furthermore, sex and gender factors also intersect with factors such as education and cultural context. Evidence suggests that the diffusion of cannabis experimentation among men appears similar to that observed with tobacco, with use beginning among men and the most educated groups first, in countries such as USA and Germany. In France, cannabis experimentation continues to be more prevalent among women with higher education [28].

Not everyone who uses cannabis transitions to cannabis use disorder (CUD). It is estimated that approximately 9% of those who initiate cannabis use will meet the criteria for cannabis use dependence. Those who initiate during adolescence have an increased likelihood (16.6%) of developing CUD [29,30]. Multiple factors have been associated with cannabis use disorder in women and men. Specifically, both frequency of use and form of cannabis used have been associated with CUD. Among females, cannabis use with strangers was more strongly related to being diagnosed with CUD according to the Diagnostic and Statistical Manual of Mental Disorders (DSM-IV) compared to males [31]. Compared to women, men have a younger age of onset for CUD [32]. Polysubstance use, trauma and violence may also be risk factors for CUD. In a US study, sexual abuse and history of alcohol use disorder were more strongly associated with 12 month CUD among females, compared to males [33]. Men with lifetime CUD were more likely than women to be diagnosed with any psychiatric disorder, any substance use disorder and antisocial personality disorder, whereas women with CUD had more mood and anxiety disorders [34].

Similar to other substance use, there is some evidence that females transition more quickly to cannabis use dependence compared to males. Studies found that women demonstrate a “telescoping effect”, meaning a shorter duration from onset of cannabis use to onset of CUD [34,35,36]. In a nationally representative sample of the U.S. population, there were no gender differences in the age at first or heavy cannabis use, age at onset of CUD, total number of episodes of cannabis abuse or dependence, or in the number of criteria met for cannabis dependence. However, the time from age at first use of cannabis to the age at onset of the CUD was shorter among women [34].

The results of studies on the subjective effects of cannabis are mixed, and seem to depend on the dose, route of administration (oral vs. smoked) and population (e.g., user vs. non user) [37]. After inhaling tetrahydrocannabinol (THC), women rated themselves as “higher” than men [38]; and reported higher ratings of cannabis as “good” and desire to “take again” compared to men [39]. Another study demonstrated women were more likely to describe cannabis as “good” at low doses, while men more likely to report the same at high doses [40]. In animal studies, female rats exhibit greater drug seeking behavior. In one study that primed rats for drug use and cues before a period of absence, females exhibited higher baseline cannabis intake during training, and reinstate responding for the cannabinoid at higher levels than males [41].

Finally, women and men report different CUD symptoms. For example, several studies report that women have worse withdrawal symptoms compared to men mostly related to gastrointestinal and mood symptoms [42,43,44,45]. Men are more likely than women to report experiencing insomnia and vivid dreams as withdrawal symptoms [45]. These findings have important implications since withdrawal symptoms correlate with relapse [46]. Moreover, in a sample of treatment-seeking adults with cannabis use disorder, women reported more co-occurring mental health issues (including lifetime panic disorder and current agoraphobia), and more days of poor physical health [45]. Although CUD is associated with poorer mental health and quality of life in both women and men, this pattern is more pronounced in women with CUD [47]. Animal studies also illustrate the impact of sex-related factors on withdrawal symptoms. Several studies show that females have slightly greater withdrawal symptoms than males [48]. After a week of daily THC treatment in Sprague–Dawley rats, Harte-Hargrove et al. observed the presence of locomotor depression in females but not males during the abstinence period [49].

## 3. Objective of the Present Study

This systematic review draws on a much broader scoping review on sex- and gender-related factors in substance use (initiation/uptake, patterns of use), effects, and prevention, treatment or harm reduction outcomes for four substances (opioids, alcohol, tobacco/nicotine and cannabis use). It also examined harm reduction, health promotion/prevention and treatment interventions and programs that include sex, gender and gender transformative elements to address each of the four substances. The methodology of the scoping review is described in full elsewhere [50].

Despite the evidence regarding sex and gender differences in, and impacts of cannabis use, little is known about sex- and gender-related factors in pharmacological interventions for cannabis dependence. Pharmacological interventions for cannabis dependence have been recently reviewed [51,52], but sex and gender factors have not been closely examined. Therefore, the purpose of this systematic review was to evaluate the effects of sex and gender factors in cannabis pharmacological interventions.

Our initial research question was:

What cannabis pharmacological interventions are available that include sex, gender and gender transformative elements and how effective are these in addressing cannabis use?

After examining the results of the original scoping review and realizing that there is a lack of examination of sex and gender factors in substance use interventions, and more specifically in cannabis pharmacological interventions, we decided to analyze the studies on cannabis pharmacological interventions that included women and men and sex-disaggregated the outcomes of the interventions for both sexes. In addition, we assessed the role of sex- and gender-based analysis in the included studies.

The research question was then updated to:

What cannabis pharmacological interventions are available that include both sexes and how effective are these in addressing cannabis use for women and men?

## 4. Methods

This systematic review was conducted in accordance with the Preferred Reporting Items for Systematic Reviews and Meta-Analyses (PRISMA) [53].

### 4.1. Search Strategy

A systematic search of the literature was undertaken to identify relevant studies published in English between 2007 and 2019 (up to fourth week of October). The following databases were used: PubMed, CINAHL, PsycINFO, and Embase. The search strategy was developed based on keywords and Medical Subject Headings (MeSH) terms. We based our search strategy on the search strategy developed for the scoping review [50] and, in addition, we also included more keywords relevant to pharmacological interventions such as “drug therapy”, “pharmacotherapy”, “pharmacology”, “cessation”, “addiction treatment” that were not included in the previous scoping review. An additional search was also completed from a recent systematic review on cannabis pharmacological interventions. Thirty-eight articles were included for the screening in this systematic review.

### 4.2. Literature Screening

**Figure 1:**
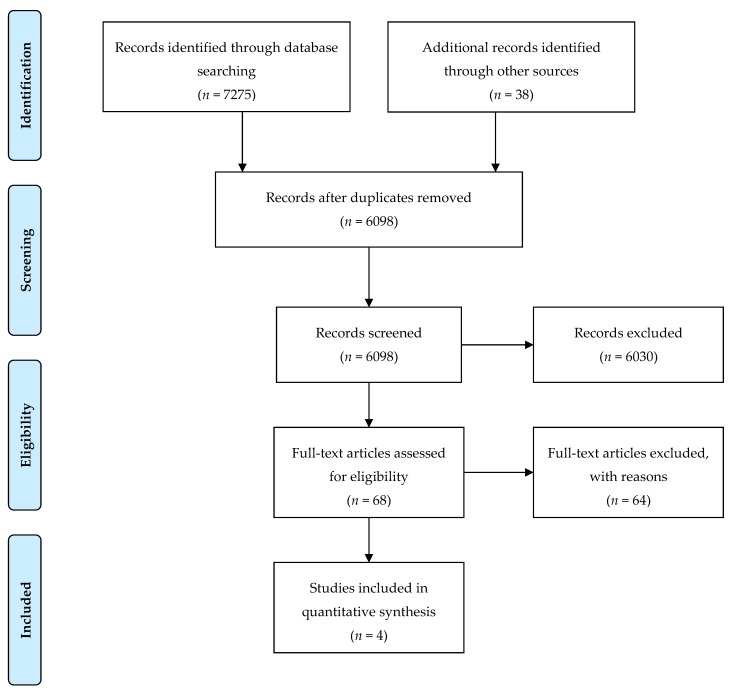
Preferred Reporting Items for Systematic Reviews and Meta-Analyses (PRISMA) diagram of study selection.

Searches in four databases resulted in *n* = 6098 unique returns. Firstly, titles and abstracts were screened by a single reviewer for relevance. Then, the full-text of the articles were obtained and reviewed by two reviewers according to the inclusion criteria. These inclusion criteria were: (a) English language articles from a selection of Organization for Economic Cooperation and Development (OECD) member countries such as Australia, Austria, Belgium, Canada, Denmark, Finland, France, Germany, Greece, Iceland, Ireland, Italy, Luxembourg, Netherlands, New Zealand, Norway, Portugal, Spain, Sweden, Switzerland, United Kingdom, United States; (b) the population of interest included: women, girls, men, boys of all ages and sociodemographic characteristics; (c) studies including pharmacotherapies that targeted cannabis use (in addition to other comorbid conditions) and presented sex-disaggregated data; (d) studies that analyzed outcomes such as cannabis abstinence or cannabis reduction; (e) randomized clinical trials. Articles were excluded if: (a) although both women and men were included in the study, outcomes of the interventions were not sex-disaggregated; (b) the study did not examine a pharmacological intervention aiming to modify cannabis use; (c) studies were conducted in a non-OECD country; (d) studies analyzed baseline characteristics of the population but the analyses are not done in relationship to the pharmacological treatment. Figure 1 provides an overview of the literature search returns, the number of articles included and excluded at each level of screening, and the final number of included articles.

### 4.3. Study Selection

The abstract screening was conducted by a single reviewer. Full papers of the included studies at this stage (*n* = 68) were then retrieved and assessed by two independent reviewers. Inter-rater reliability was calculated, and the overall kappa was 0.78. Differences between the reviewers in the inclusion of articles were resolved through discussion and consensus was reached.

### 4.4. Data Extraction

Data regarding the following information was extracted by one reviewer from the four papers included in this systematic review: (1) study details (authors and year of publication); (2) aim of the study; (3) study design; (4) country of study; (5) setting of the study; (6) details on recruitment; (7) inclusion and exclusion criteria; (8) method of allocation to intervention/control; (9) details regarding the intervention; (10) sample size and demographics; (11) baseline comparisons; (12) outcomes; (13) details on the sex, gender or diversity analysis; (14) follow up periods; (15) methods of analysis; (16) results; (17) results regarding the sex, gender or diversity based factors in findings; (18) attrition details; (19) study limitations; (20) evidence gaps and/or recommendations for future research.

### 4.5. Sex- and Gender-Based Analysis in the Included Studies

Research can incorporate sex- and gender-based analysis in several ways. Hammarström presented a tool that researchers might use when developing gender research [54]. Although Hammarström [54] does not employ the term “sex- and gender-based analysis”, in this paper we used the concept sex- and gender-based analysis as in the scoping review conducted by McCarthy et al. [55]. The authors reviewed 458 articles on pharmacy practice research and found that only six studies mention any information related to sex and gender considerations and only three were classified as SGBA according to Hammarström’s model [55]. Table 1 presents the classification based on Hammarström’s typology [54]. For the sex- and gender-based analysis of the included articles, we examined the following characteristics:

1. *Use of sex and gender in the aim and research questions*: were sex and gender included in the aim of the study or explicitly mentioned in the research question and the study design?
2. *Study design and reporting results*: how were the outcomes analyzed and reported in relation to sex and gender?
3. *Interpretation of sex/gender findings*: how were findings related to sex and gender included in the interpretation of the data?
4. *Intentional and accurate use of language*: were the terms sex and gender used intentionally and appropriately by the authors of the study?

## 5. Results

### 5.1. Included Studies

Four randomized controlled trials involving 623 participants met the inclusion criteria for this review [56,57,58,59]. Characteristics of the studies are described in Table 2. In total, 316 participants received the intervention while 307 participants received placebo. The number of women included in the studies oscillated between 16 [58] and 86 [57]. Disaggregating by sex, 170 women and 453 men were included in the randomized controlled trials and 82 women and 234 men received the pharmacological intervention. In the placebo group there were 88 women and 219 men. In addition to the pharmacological intervention and placebo, some form of psychological intervention was offered in all included studies.

Several medications with different mechanisms of action were applied in the studies included in this review. Cornelius et al. [57] examined the role of fluoxetine while McRae-Clark et al. [58] used vilazodone. Both medications are selective serotonin reuptake inhibitors. The effect of buspirone, a serotonin 5-HT1A partial agonist, was explored by McRae-Clark et al. [59]. Lastly, Gray et al. [57] examined the effect of N-acetylcysteine, a supplement promoting glutamate release and modulating N-methyl-D-aspartate (NMDA).

All studies were undertaken in outpatient settings. In one study, the scheduled duration for the clinical trial was 8 weeks [58] while in the three other studies, it was 12 weeks [56,57,59]. The four selected studies were all conducted in the USA. The mean age of participants was between 16.64 [56] and 29.8 years [57]. Three studies included young adults [57,58,59] and one study targeted adolescents [56]. In one study, participants had comorbid major depression and cannabis use disorders [56]. The other three studies excluded people with psychiatric conditions.

### 5.2. Sex-Disaggregated Outcomes

In one of the included articles, sex was not a significant predictor of cannabis abstinence, and there was no sex-by-treatment interaction [57]. Females showed a greater improvement with time on depressive symptoms (*F* = 5.01, *p* = 0.028) and DSM cannabis abuse criteria count than males (*F* = 4.22, *p* = 0.044) [56]. In a study using buspirone McRae-Clark et al. (2015) [59] found that UCTs were negative in 8.7% of buspirone and 4.5% of placebo of male participants. In females, 2.4% of buspirone participant UCTs were negative and 12.9% of placebo; although the difference was not statistically significant (*p* = 0.007). Regarding the creatinine adjusted cannabinoid levels, there was a sex by treatment interaction indicating that for males, those randomized to buspirone treatment had significantly lower creatinine adjusted cannabinoid levels as compared to those randomized to placebo. For females, those randomized to placebo had lower creatinine adjusted cannabinoid levels compared to those randomized to buspirone [59]. Examining the effect of vilazodone, McRae-Clark (2016) found that males had significantly lower creatinine-adjusted cannabinoid levels and a trend for increased negative urine cannabinoid tests compared to females [58].

### 5.3. Sex- and Gender-Based Analysis of the Included Studies

The assessments of the role of sex- and gender-based analysis in the included studies is presented in Table 3. While Cornelius et al. and Gray et al.´s studies were classified in the sex/gender differences category, McRae-Clark et al. (2015) [59] and McRae-Clark et al. (2016) [58] were categorized as SGBA (see Table 3). Based on the categories that were analyzed, the results are as follows:

1. *Aim and research questions*: The four studies included sex/gender in the study design or the reporting. However, none of the studies included sex or gender in their major research question.

2. *Reporting sex/gender in the results*: In Cornelius et al.´s study [56] on the effects of fluoxetine in adolescents and young adults with comorbid depression and cannabis use dependence, sex by time was analyzed for the outcomes of the study (number of days participants used cannabis in past month; DSM cannabis dependence count; DSM CUD total count - DSM dependence + abuse symptoms). The authors also examined whether abstinence rates differed across sex [56]. Although Gray et al. did not find statistically significant results, they examined whether sex was a predictor of cannabis abstinence, and whether there was a sex-by-treatment interaction [57]. McRae-Clark (2016) used sex as one of the randomization variables in addition to the presence or absence of anxiety or depressive disorders [58]. Sex and sex by treatment group interactions were added to examine the effect of gender on the primary and secondary efficacy outcomes in a randomized clinical trial that tested the efficacy of vilazodone, a selective serotonin receptor inhibitor and partial 5-HT1A agonist, for treatment of cannabis dependence [58]. McRae-Clark et al. also conducted a sex- and gender-based analysis since they used sex as a stratified randomization variable [59]. Sex was analyzed in relationship to the negative UCTs and cannabinoid levels in this study that examined the efficacy of buspirone for participants with cannabis use dependence [59].

3. *Interpretation of Sex/Gender findings*: Cornelius et al. did not report their findings related to sex and/or gender in the discussion section [56]. Gray et al. did not discuss any aspects of sex or gender, likely because their results were not statistically significant [57]. The differences reported in the results section are interpreted and explained in McRae-Clark et al. (2015) [59] and McRae-Clark et al. (2016) [58]. McRae-Clark et al.’s study, which featured sex or gender in their research question, provided a comprehensive discussion of their interpretation of the impact of sex and gender in their findings [59]. In this study, the authors acknowledged that this is the first study to demonstrate a sex difference in response to a pharmacological treatment for cannabis dependence. The authors highlighted the importance of including gender in the development and evaluation of new treatments for addictive disorders [59]. However, they did not specify what sex or gender-related factors could be considered for the development and evaluation of new treatments for addictive disorders. McRae-Clark et al.’s (2016) study suggests that women with CUD might have more problems in achieving cannabis cessation compared to men with CUD [58]. Their findings are related to sex and gender in the discussion. They also note that their analyses of sex differences might have been underpowered, and they mention that women are underrepresented in pharmacological trials calling for higher representativity of women in future studies.

4. *Intentional and accurate use of terminology*: None of the included studies define sex and gender. Cornelius et al. use only the term sex and they do not mention gender [56], while Gray et al. used sex and gender interchangeably [57]. For example, in the sociodemographic table the authors use “gender” and throughout the paper they mentioned “sex”. McRae-Clark et al. and McRae-Clark et al. used “gender” throughout the article though the study is in fact measuring sex although they also employ the terms females and males and women and men at the same time [58,59]. All four articles included in this systematic review lacked accuracy in the application of the concepts of sex and gender. Not even the articles that were categorized as applying a sex- and gender-based analysis in their studies used intentional and accurate terminology throughout the articles.

## 6. Discussion

In this systematic review on sex- and gender-related factors in cannabis pharmacological interventions, there was a paucity of studies that sex-disaggregated outcomes for women and men or analyzed the sex- or gender-related factors in the interventions. Although overall the findings showed that the pharmacological interventions analyzed in the studies (fluoxetine, vilazodone, buspirone, N-acetylcysteine) are not effective for treating CUD, three of the four included studies found different results for women and men. Of the three studies, one showed that females demonstrated a greater improvement with time on the depressive symptoms and DSM cannabis abuse criteria count than males [56]. The other two studies suggest that women have worse results than men in cannabis pharmacological interventions [58,59].

The lack of reported sex-disaggregated results does not mean that there are no differences or similarities between women and men. However, it is not possible to accurately interpret these results. Given the emergent evidence of sex- and gender-related factors in cannabis research [42,43], sex- and gender-related factors may intervene in the efficacy of cannabis pharmacological interventions. As in the case of smoking cessation treatment, demonstrating that women have more difficulty maintaining long-term abstinence than men [60], two of the four included studies showed that women have worse outcomes when examining the efficacy of buspirone [59] and vilazodone [58].

Even though the included studies did not find a greater efficacy of the pharmacological intervention, two of the four studies found that women had better results in the placebo group while men had better results in the pharmacological intervention group [58,59]. The different mechanisms generating the placebo effect between women and men are not well understood. However, preliminary findings suggest that sex- and gender-related factors might also be intervening in the placebo effect [61].

Although two of the included studies described the integration of aspects of sex into research questions, analysis, reporting of findings and discussion, there is an overall lack of comprehensive integration and analysis of sex and gender in these randomized controlled trials. These findings are consistent with those found by Welch et al. (2017) examining the use of sex and gender considerations in 100 Canadian-led or funded RCTs [6]. This study showed that 98% of studies included sex in the description of sociodemographic characteristics of the participants and only 6% conducted a subgroup analysis across sex and 4% reported sex-disaggregated data. Even in those RCTs that included females, most of the studies did not sex-disaggregate the outcomes [6].

Studying the effect of sex- and gender-related factors in cannabis pharmacological interventions is challenging and there is still an overall lack of research on sex, gender and cannabis. To determine sex- and gender-related factors in pharmacological interventions for cannabis use, researchers urgently need to fill this void. The preliminary findings show that women might not benefit from certain pharmacological interventions. Including and reporting sex- and gender-related factors might contribute to better determine the effectiveness of pharmacological interventions for both women and men and tailor treatment for all individuals.

In the included studies, the terms “sex” and “gender” were used in an inconsistent way and there were no definitions provided for these terms. Three of the included studies used “sex” and “gender” interchangeably. Throughout the studies, authors used “male/female” and “women/men” and the use of “gender” was inaccurate. These findings are consistent with results from a study on Campbell and Cochrane systematic reviews [62]. Petkovic et al. (2018) found that reporting in systematic reviews is inadequate [62]. None of the studies in our systematic review included gender diverse populations or other gender considerations. Findings from a scoping review on how gender norms, roles and relations impact cannabis use patterns showed that there is a complex relationship between substance use and gender norms. While certain feminine and masculine norms might be protective, there are others that might be linked with greater risk of developing cannabis use dependence [50].

This systematic review has limitations. Since sex and gender are not often examined in pharmacological interventions for cannabis use, our results are limited. This is reflected in the small number of studies that met the inclusion criteria, and therefore, what we could draw from for interpretation. Our search strategy was designed taking into account that there is a growing body of literature that focuses on sex- and gender-related factors and we conducted searches using sex- and gender-related keywords [63]. However, since the use of sex and gender terms are not used in pharmacological interventions for cannabis use, we reviewed references from a recent systematic review [52] and screened those that were not captured by our search strategy. Sex and gender factors might have been tested in many other studies but not reported. We did not contact authors for further details on sex- and gender-based analysis, methods or results. Although we intended to apply the Feminist Quality Appraisal Tool [64] to analyze the ways in which gender is addressed in the included studies, the lack of deeper gender analysis did not support it. We did not perform a quality assessment of the studies since our aim was to examine the role of sex- and gender-related factors and the uptake of sex- and gender-based analysis. The included articles were assessed in two previous systematic reviews that examined the effectiveness of pharmacotherapies for cannabis dependence [51,52].

## 7. Conclusions

This systematic review aimed to examine the treatment outcomes in cannabis pharmacological interventions for women and men. In addition, it analyzed the uptake of sex- and gender-based analysis in pharmacological interventions for cannabis use. Despite the increasing evidence showing that sex and gender factors intervene in patterns of cannabis use, health effects and biological mechanisms, we found only four articles that sex-disaggregated the outcomes for both sexes on CUD treatment. Taking into account the poor uptake of sex- and gender-based analysis, future research should consider more consistent and disciplined integration of sex and gender in cannabis pharmacological interventions in order to improve outcomes for all individuals experiencing CUD.

## Figures and Tables

**Table 1:** Sex- and gender-based analysis in health research.

Research Phase	Model 1: Sex/Gender Differences	Model 2: Sex and Gender-Based Analysis (SGBA)	Model 2(a): SGBA+	Model 3: Intersectional Approach
Research question	Sex/gender included, but not primary focus of study. Sex/gender included in the study design or the reporting but are not specifically stated in the research question or aim of the study.	Specific questions related to sex/gender. Looking for sex/gender differences, or the impact of sex/gender an explicit aim of the study or stated research question.	Specific questions related to sex/gender, and additional subgroups/identities included. Research question includes sex/gender and other factors such as race, age, sexual orientation, etc.	Specific questions related to sex/gender, and additional subgroups/identities included. Research question includes sex/gender and other factors such as race, age, sexual orientation, etc.
Data analysis and reporting of findings	Disaggregation by sex/gender; sex as confounder/controlled for (e.g., included in a model). Data related to the outcomes is reported for different sex/gender groups or sex/gender is controlled for in the analysis.	Sex/gender as analysis category Beyond reporting results by different sex/gender group, there is testing of significance between gender groups in relation to the outcomes of the study.	Sex/gender as analysis category; other factors included (e.g., race, SES). There is testing of significance between sex/gender groups in relation to the outcomes of the study and related to other factors such as race, ethnicity, age, etc. But as sperate analysis, not combined into one analysis. Must be beyond reporting demographic characteristics of a sample.	Multi-faceted analysis of multiple factors. More than one factor is included in the same analysis (e.g., comparing young and old white and Hispanic men, to the same 4 groups of women).
Interpretation of sex/gender findings	Findings related to sex and gender are not necessarily included in the interpretation of the data. Differences reported are not necessarily explained.	Findings related to sex and/or gender are reported in the discussion/conclusion. The differences reported in the results section are interpreted and explained.	Findings related to sex and/or gender are reported in the discussion/conclusion in relationship to at least another factor.	Findings related to sex and/or gender are reported in the discussion/conclusion in relationship to other factors such as race, age, etc. The differences reported in the results section are interpreted and explained.
Use of language	Not dependent on specific aim, design/results and interpretation.	Not dependent on specific interpretation and use of language.	Not dependent on specific interpretation and use of language.	Not dependent on specific interpretation and use of language.

Adapted from: Hammarstrom (2007) [54]; McCarthy et al. (2017) [55].

**Table 2:** Characteristics and findings of included studies.

Cornelius et al. (2010) [56]	Characteristics and Findings of Included Studies
Study design	Randomized controlled trial
Participants	Recruitment: Through referrals from the Western Psychiatric Institute and Clinic (WPIC) treatment programs and by responding to newspaper, radio, and bus advertisements. Setting: Outpatient clinic, Pittsburgh, USA. Scheduled for 12 weeks. Participants: In total, 70 participants between 14 and 25 years of age at baseline and comorbid presence of both a current CUD (using DSM-IV) and a current major depressive disorder (MDD). Exclusion criteria: Diagnosis of bipolar disorder, schizoaffective disorder, or schizophrenia; subjects with hyper- or hypothyroidism, significant cardiac, neurological, or renal impairment, and significant liver disease; substance abuse or dependence other than alcohol abuse or dependence, nicotine dependence, or cannabis abuse; any history of intravenous drug use; pregnancy, inability or unwillingness to use contraceptive methods, and an inability to read or understand study forms. Sample size: Intervention, 34; placebo: 36. Demographics: Mean age 21.1 years ±2.4 years; 61% male; 56% Caucasian, 37% African-American. In total, 94% cannabis dependent, using on average of 76% of days in prior month; 20 participants met diagnostic criteria for alcohol dependence; seven for alcohol abuse and 16 reported a history of an antidepressant medication in the moth prior to recruitment.
Interventions	Intervention: In total, one capsule of 10 mg of fluoxetine for 2 weeks and increased to two capsules of 10 mg of fluoxetine. Placebo: In total, one capsule of 10 mg of placebo and after 2 weeks, two capsules of 10 mg of placebo. The low dose was used to maximize the safety and minimize the risk of medication side effects. In total, nine sessions of cognitive behavior therapy (CBT) for depression and CUD, and motivation enhancement therapy (MET) for CUD.
Outcomes	Severity of abuse or dependence (cannabis and alcohol), number of days of cannabis use, quantity and frequency, number completing the treatment Timeline follow-back method (TLFB) for the cannabis use behaviors and other substance use behaviors; Hamilton Rating Scale for Depression (HAM-D-27) for observer-rated depressive symptoms; Beck Depression Inventory (BDI) for participant-rated depressive symptoms; Number of drinks per drinking day, the number of drinking days, number of heavy drinking days (defined as greater than or equal to four drinks per day for women and five for men); Side Effects Questionnaire for Children and Adolescent for the side effects during each assessment throughout the course of the clinical trial.
Findings	The group that received fluoxetine did not have better cannabis or depressive than the group that received placebo. The improvement of the depressive symptoms and decrease of number of days of cannabis use may have resulted either from the psychosocial therapy or the natural course of the disorders.
Gray et al. (2017) [57]	
Study design	Randomized controlled trial
Participants	Recruitment: Community media advertisements. Setting: Outpatient, six sites within the National Drug Abuse Treatment Clinical Trials Network, USA. Scheduled duration 12 weeks. Participants: In total, 302 treatment-seeking adults ages 18–50 with CUD and submitting a positive Urine cannabinoid testing UCT during the initial screening visit. Exclusion criteria: Individuals with acutely unstable medical or psychiatric disorders, DSM-IV-TR substance dependence aside from cannabis or tobacco, contraindications for N-acetylcysteine (NAC) treatment, or recent synthetic cannabinoid use. Sample size: Intervention, 153; placebo, 149. Demographics: Mean age 29.8 years ±8.74 years; 71.5% male; 58.3% White; 27.8% Black or African-American. Mean cannabis use 26.0/30 days at baseline.
Interventions	Intervention: In total, two capsules of 600 mg of United States Pharmacopeia grade NAC powder (twice-daily dose). Placebo: In total, two capsules of 600 mg of placebo (twice per day). Riboflavin 25 mg was added to all capsules (100 mg/day total) as a biomarker for medication adherence. All participants received contingence management twice weekly during treatment. Medical management.
Outcomes	Urine specimens were collected at baseline, twice weekly throughout treatment, at end-of-treatment. UCT at post-treatment follow-up. Medication adherence included taking ≥80% of prescribed study medication per study week, confirmed by urine riboflavin level >1500 ng/mL. Adverse effects at each study visit.
Findings	No statistically significant differences between the NAC and placebo groups in cannabis abstinence. In the NAC group, 22.3% of urine cannabinoid tests were negative compared to 22.4% in the placebo group. Exploratory analysis within medication-adherent subgroups revealed no significant differential abstinence outcomes by treatment group.
McRae-Clark et al. (2015) [59]	
Study design	Randomized controlled trial.
Participants	Recruitment: Media and internet advertisements. Setting: Outpatient. Scheduled duration 12 weeks. Participants: In total, 175 participants between 18 and 65 years of age and met DSM-IV criteria for current cannabis dependence. Exclusion criteria: current dependence on any other substance (with the exception of caffeine and nicotine), history of psychotic, bipolar or eating disorder, current suicidal or homicidal risk, current major depression, current treatment with psychoactive medication (with the exception of stimulants and non-benzodiazepine sedative/hypnotics), major medical illness or disease, significant cognitive impairment, hypersensitivity to buspirone or other product component, current consumption of substances that inhibit or induce CYP3A4, and pregnancy, lactation or inadequate birth control. Sample size: intervention, 88; placebo, 87. Demographics: Mean age 24.00 years (23.1-25 years); 76.6% male; 64% Caucasian.
Interventions	Intervention: Dosage initiated at 5 mg buspirone or placebo twice daily and increased by 5–10 mg every three to four days as tolerated, to a maximum dose of 60 mg daily for 12 weeks. Placebo: Up to 60 mg of placebo. Adjunctive motivational enhancement therapy sessions (MET) during the first four weeks of the treatment period.
Outcomes	Semi-quantitative urine cannabinoid tests (UCTs) for cannabinoids administered at screening and weekly throughout the study. Proportion of negative urine test during treatment. Point prevalence of abstinence by urine test at the end of the treatment Number of reporting adverse events.
Findings	No differences of UCTs and the weekly creatinine adjusted cannabinoid levels between the two groups. Although participants in both groups reduced their cannabis craving over the course of the study, there were no differences between the buspirone and placebo groups. However, participants who attained abstinence from cannabis reported less cannabis craving.
McRae-Clark et al. (2016) [58]	
Study design	Randomized controlled trial
Participants	Recruitment: Media and internet advertisements. Setting: Outpatient, 8 weeks. Participants: In total, 76 participants between 18 and 65 years of age and CUD. Exclusion criteria: current dependence on any other substance (exception caffeine and nicotine), history of psychotic, bipolar, or eating disorder, current suicidal or homicidal risk, current treatment with psychoactive medication (exception stimulants and non-benzodiazepine sedative/hypnotics) or CYP3A4 inhibitors, major medical illness or disease, pregnancy, lactation, or inadequate birth control, patients that would be unable to comply with study procedures or assessments. Sample size: Intervention, 41; placebo, 35. Demographics: Mean age 22.2 (21.3–23.1) years; 79% male; 54.8% Caucasian.
Interventions	Intervention: In total, 10 mg daily dose of Vilazodone tablets provided by Forest Pharmaceuticals for 7 days, increased to 20 mg daily for 7 days, followed by 40 mg daily as tolerated. Placebo: In total, 10 mg daily dose of placebo tablets for 7 days, increased to 20 mg daily for 7 days, followed by 40 mg daily. Both groups received three adjunctive motivational enhancement therapy sessions (MET). First session, prior to medication initiation. Second session, approximately 1 week later. Third session, week 4.
Outcomes	Quantitative urine cannabinoid tests (UCTs) for cannabinoids administered at screening and weekly throughout the study. Self-report cannabis use measured by TLFB (Time-Line Follow-Back). Marijuana Craving Questionnaire (MCQ) for levels of cannabis craving. Adverse effects assessed weekly. Medication compliance by weekly patient report. Proportion of scheduled visits attended.
Findings	The vilazodone group did not show greater efficacy when compared to the placebo group on cannabis use outcomes. Participants in both groups reported lower cannabis use with no differences between the two groups.

**Table 3:** SGBA applied to cannabis pharmacological interventions.

Authors	Publication Date	SGBA Categorization	Sex/Gender in the Research Question	Results	Interpretation of Sex/Gender Findings	Use of Terminology	Findings Related to Sex and Gender
[56]	2010	Sex/Gender Differences	No	Sex by time was analyzed in relation to the outcomes.	No	Use only sex	Females showed a greater improvement with time on the depressive symptoms and DSM cannabis abuse criteria count than males.
[57]	2017	Sex/Gender Differences	No	Examined whether sex was a predictor of cannabis abstinence, and whether there was a sex-by-treatment interaction.	No	Sex and gender used interchangeably	Sex was not a significant predictor of cannabis abstinence, and there was no sex-by-treatment interaction.
[59]	2015	SGBA	No	Sex was used as a randomized stratification variable. Sex was analyzed in relationship to the negative UCTs and cannabinoid levels.	Yes	Sex and gender used interchangeably	In males, 8.7% of buspirone participant UCTs were negative and 4.5% of placebo UCTs were negative. In females, 2.4% of buspirone participant UCTs were negative and 12.9% of placebo; although the difference was not statistically significant (*p* = 0.007). There was a sex by treatment interaction for the creatinine adjusted cannabinoid levels: for males, those randomized to buspirone treatment had significantly lower creatinine adjusted cannabinoid levels as compared to those randomized to placebo; for females, those randomized to placebo had lower creatinine adjusted cannabinoid levels compared to those randomized to buspirone.
[58]	2016	SGBA	No	Sex was used as a variable for randomization. Sex and sex by treatment group interactions were analyzed.	Yes	Sex and gender used interchangeably	Men had significantly lower creatinine-adjusted cannabinoid levels and a trend for increased negative urine cannabinoid tests than women. There were no sex differences regarding the self-reported frequency and amount of cannabis use; nor significant interactions between sex and treatment. Male participants randomized to vilazodone showed a reduction in the Purposefulness subscale of the MCQ; it did not happen for females.
